# Methicillin-Resistant *Staphylococcus aureus* Infection and Hospitalization in High-Risk Patients in the Year following Detection

**DOI:** 10.1371/journal.pone.0024340

**Published:** 2011-09-16

**Authors:** Susan S. Huang, Virginia L. Hinrichsen, Rupak Datta, Laura Spurchise, Irina Miroshnik, Kimberly Nelson, Richard Platt

**Affiliations:** 1 Division of Infectious Diseases and Health Policy Research Institute, School of Medicine, University of California Irvine, Irvine, California, United States of America; 2 Channing Laboratory, Brigham and Women's Hospital and Harvard Medical School, Boston, Massachusetts, United States of America; 3 Department of Population Medicine, Harvard Medical School and Harvard Pilgrim Health Care Institute, Boston, Massachusetts, United States of America; Los Angeles Biomedical Research Institute, United States of America

## Abstract

**Background:**

Many studies have evaluated methicillin-resistant *Staphylococcus aureus* (MRSA) infections during single hospitalizations and subsequent readmissions to the same institution. None have assessed the comprehensive burden of MRSA infection in the period after hospital discharge while accounting for healthcare utilization across institutions.

**Methodology/Principal Findings:**

We conducted a retrospective cohort study of adult patients insured by Harvard Pilgrim Health Care who were newly-detected to harbor MRSA between January 1991 and December 2003 at a tertiary care medical center. We evaluated all MRSA-attributable infections associated with hospitalization in the year following new detection, regardless of hospital location. Data were collected on comorbidities, healthcare utilization, mortality and MRSA outcomes. Of 591 newly-detected MRSA carriers, 23% were colonized and 77% were infected upon detection. In the year following detection, 196 (33%) patients developed 317 discrete and unrelated MRSA infections. The most common infections were pneumonia (34%), soft tissue (27%), and primary bloodstream (18%) infections. Infections occurred a median of 56 days post-detection. Of all infections, 26% involved bacteremia, and 17% caused MRSA-attributable death. During the admission where MRSA was newly-detected, 14% (82/576) developed subsequent infection. Of those surviving to discharge, 24% (114/482) developed post-discharge infections in the year following detection. Half (99/185, 54%) of post-discharge infections caused readmission, and most (104/185, 55%) occurred over 90 days post-discharge.

**Conclusions/Significance:**

In high-risk tertiary care patients, newly-detected MRSA carriage confers large risks of infection and substantial attributable mortality in the year following acquisition. Most infections occur post-discharge, and 18% of infections associated with readmission occurred in hospitals other than the one where MRSA was newly-detected. Despite gains in reducing MRSA infections during hospitalization, the risk of MRSA infection among critically and chronically ill carriers persists after discharge and warrants targeted prevention strategies.

## Introduction

It is well known that MRSA acquisition confers a high risk of infection during the same hospitalization. Studies performed in the past twenty years indicate that approximately 11–20% of patients who newly acquire MRSA develop subsequent infection during hospitalization [1]–[2]. These risks are even higher in intensive care unit (ICU) patients, with 24–27% of colonized patients developing infection during hospitalization [3]–[5]. Increased risks have also been reported in other hospitalized populations such as patients with chronic ulcers [6].

However, less is known about the long-term risks of MRSA acquisition. This is important because post-discharge medical care is often provided through a variety of methods, such as nursing homes, rehabilitation centers, and home health care services, and hospital length of stay continues to shorten. Recent data indicate that over half of hospitalizations in the United States are 3 days or less [7]. Thus, despite gains in reduction of healthcare-associated infections during hospital stays [8]–[9], studies are needed to evaluate the full burden of MRSA acquisition after hospital discharge.

The need to understand post-acquisition MRSA risks is magnified by widespread initiatives to identify MRSA carriers. As of 2011, fifteen states have enacted legislation involving screening, reporting, or other laws targeting MRSA carriers [10]. Nevertheless, once MRSA carriers are identified, little is done to prevent infection in patients harboring MRSA. Most efforts focus on contact precautions and room isolation to prevent MRSA transmission to other inpatients [11]–[12]. Furthermore, limited data that do exist on long-term risks suggest that once carriers are discharged, MRSA infection and readmission commonly occurs shortly after discharge and remains elevated up to 12–18 months following discharge [13]–[14]. Additional data on MRSA sequelae in high-risk patients may not only enhance targeted interventions and future studies seeking to mitigate MRSA morbidity and mortality in this group, but also inform current multi-state mandates to screen high-risk hospitalized patients.

We sought to assess the risk of subsequent MRSA infection, hospitalization and death among high-risk inpatients newly-detected to harbor MRSA at a tertiary hospital to understand the magnitude of MRSA burden in a sick cohort. To reduce the complexity of potential differential virulence due to community-acquired versus hospital-associated MRSA strains, we studied a cohort during a time period when healthcare-associated MRSA was the dominant clone at a single institution.

## Methods

We conducted a retrospective cohort study to evaluate the frequency of subsequent MRSA infection and death among patients newly-detected to harbor MRSA. We identified a cohort of adult patients insured by Harvard Pilgrim Health Care who were newly-detected to have a MRSA-positive culture between January 1, 1991 and December 31, 2003 at Brigham and Women's Hospital, a 750-bed tertiary care academic medical center in Boston, MA. This study period corresponds to a time window when genetic testing (pulse field gel electrophoresis) was being performed on a representative sample of hospital MRSA strains, and nearly all strains were confirmed to be healthcare-associated clones (Yokoe D. Personal communication). In addition, nares surveillance testing for MRSA was not routinely performed during the study period.

Harvard Pilgrim Health Care is a full-service health benefits company serving patients throughout New England with a network of over 135 hospitals and 28,000 physicians [15]. Patients insured by Harvard Pilgrim Health Care were retained in the cohort if they had no prior evidence of MRSA-positivity based on microbiology and infection control records at Brigham and Women's Hospital. Patients reporting a history of MRSA-positivity from other institutions were excluded. We assessed the risk of subsequent MRSA infection and death among these newly-detected MRSA carriers by evaluating medical records from the admission in which MRSA was detected plus all subsequent inpatient medical facilities in the year following discharge. Newly-detected MRSA carriage represented colonization or infection by definition. This study was approved by the Institutional Review Boards of Brigham and Women's Hospital and Harvard Pilgrim Health Care.

### Description of Procedures

For outcomes, all inpatient admissions in the year following MRSA detection were identified by date and hospital name, and discharge summary and microbiologic culture data were requested. If evidence of MRSA-positive cultures was found during inpatient hospitalizations, full medical records were obtained. Outpatient MRSA cultures were not assessed. Medical records were reviewed to identify the source of the MRSA-positive culture at the time of new detection and determine whether the positive culture represented colonization or infection based on Centers for Disease Control and Prevention criteria [16]. All subsequent MRSA isolates within a one-year period from the time of new detection were evaluated for evidence of discrete and unrelated MRSA infection by reviewers experienced in applying Centers for Disease Control and Prevention criteria for infection detection. All infection criteria were additionally verified by an infectious diseases physician. Subsequent MRSA infections were described according to (1) the number of days between new detection and the onset of infection, (2) the type of infection, and (3) whether the infection was associated with MRSA bacteremia. Infection type was assigned according to the primary source of infection based on Centers for Disease Control and Prevention criteria [16].

For descriptive purposes, we obtained demographic, comorbidity and healthcare utilization data for all patients from Harvard Pilgrim Health Care insurance records. Healthcare utilization included acute hospital admissions, sub-acute hospital admissions, admissions to an intensive care unit, emergency room visits, outpatient visits, surgical and non-surgical procedures in the year before and after MRSA detection. Comorbidities were identified using diagnoses of heart disease, asthma, diabetes mellitus, end-stage renal disease, end-stage liver disease, and noncancer immunocompromised states based on *International Classification of Diseases, Ninth Revision* codes from healthcare claims data in the year prior to MRSA detection. Identified comorbidities were verified through medical record review. For all patients newly-detected with MRSA during an inpatient stay, we also determined their preadmission location, hospital discharge disposition, intensive care unit admission, and length of hospital stay.

We assessed patient characteristics as the proportion of total patients with the specified attribute. We determined the proportion of patients who were colonized or infected at the time of detection and the proportion of patients who developed any subsequent infection in the year following MRSA detection. We evaluated all-cause mortality among patients newly-detected with MRSA using both Harvard Pilgrim Health Care insurer records and the Massachusetts Mortality File. We also assessed whether deaths were associated with MRSA infection at the time of detection or during the year following detection. If deaths were associated with MRSA, we further determined whether deaths were attributable to MRSA. Death was deemed to be associated with MRSA if MRSA bacteremia was found within 7 days of death or if there was active MRSA infection at the time of death as previously described [2], [14]. Death was additionally deemed to be attributable to MRSA if the above criteria were met and there was no alternative cause of death [2], [14].

### Statistical Methods

Potential predictors of subsequent MRSA infection among patients newly-detected with MRSA were assessed using χ^2^ tests. Variables significant in bivariate testing at a level of α<0.2 were entered into a logistic regression model. Final model variables were retained at α = 0.05.

## Results

We identified 591 patients insured by Harvard Pilgrim Health Care with an MRSA-positive culture between January 1, 1991 and December 31, 2003 at the Brigham and Women's Hospital who had no prior evidence of MRSA-positivity. In keeping with rising MRSA prevalence across the U.S., 22% (N = 123) of the cohort was identified from 1991–1995 (N = 189), 31% from 1996–1999, and 47% (N = 279) from 2000–2003. Patient characteristics are summarized in Table 1. The mean age of patients at the time of new detection was 62 years (median age, 67 years). Most individuals were inpatients at the time they were newly-detected with MRSA, and 40% (230/576) had an MRSA-positive culture within 2 calendar days of admission to Brigham and Women's Hospital. The mean length of hospital stay among inpatients was 22 days (median duration, 16 days).

**Table 1:** Characteristics of Patients at Time of New Detection with Methicillin-Resistant *Staphylococcus aureus* (MRSA).

Characteristic	No. (%) of Patients (N = 591)
**Male Gender**	337 (57%)
**Age**	
18–34 years	28 (5%)
35–44 years	42 (7%)
45–54 years	73 (12%)
55–64 years	125 (21%)
65–74 years	149 (25%)
75–84 years	147 (25%)
≥85 years	27 (5%)
**Comorbidity**	
Heart Disease	349 (59%)
Diabetes Mellitus	154 (26%)
Asthma	106 (18%)
Immunocompromised, Noncancer	77 (13%)
End-Stage Renal Disease	41 (7%)
End-Stage Liver Disease	30 (5%)
**Healthcare Utilization in Year Prior to MRSA Detection** *	
Any Hospitalization	476 (81%)
Acute Care Hospital	467 (79%)
Intensive Care Unit Stay	183 (31%)
Emergency Department Visit	435 (74%)
Non-Surgical Procedures	409 (69%)
Surgery	357 (60%)
Skilled Nursing Facility or Rehabilitation Stay	229 (39%)
None of the Above	53 (9%)
Outpatient Visit	563 (95%)
**Healthcare Location at Time of New Detection** †	
Inpatient	576 (97%)
Outpatient	15 (3%)
**Pre-Admission Location** † ^, ^ ‡	
Home	372 (65%)
Rehabilitation Facility	53 (9%)
Skilled Nursing Facility	31 (5%)
Hospital Transfer	120 (21%)
**Discharge Disposition** † ^, ^ ‡	
Home	195 (33%)
Rehabilitation Facility	233 (39%)
Skilled Nursing Facility	41 (7%)
Hospital Transfer	13 (2%)
Deceased§	94 (16%)

*Not including admission in which MRSA was newly-detected.

† Evaluation for admission where patient was newly-detected with MRSA.

‡ n = 576.

§ Deaths refer to all-cause mortality during admission where patient was newly-detected with MRSA.

Of the 591 patients who were newly-detected to harbor MRSA, 138 (23%) were colonized and 453 (77%) were infected at the time of entry into the cohort. Among those patients who were colonized, common sources of MRSA colonization included sputum (53% of patients), nares (24%), and wounds (9%). Infections at the time of MRSA detection most often included pneumonia (41% of patients) and skin and soft tissue (19%) infections, with 18% of detection infections associated with bacteremia.

Overall, 196 patients (33%) developed 317 discrete and unrelated infections in the year after detection of MRSA carriage (Table 2). The mean time to first MRSA infection following new detection was 68 days (median, 32 days). Most patients experienced subsequent MRSA pneumonia, skin and soft tissue, and primary bloodstream infections. Of all 317 subsequent MRSA infections, 26% were associated with bacteremia. There was no difference in the risk of subsequent infection by year (χ^2^ = 7.48, p = 0.82) of cohort entry. There was also no difference in infection risk according to whether patients were colonized (42/138, 30%) or infected (154/453, 34%) at the time of new detection (χ^2^ = 6.77, p = 0.47). Among inpatients, the risk of subsequent infection differed significantly according to whether patients were admitted from another hospital (40%, 48/120), home (33%, 122/372), or a rehabilitation or skilled nursing facility (23%, 19/84) (χ^2^ = 6.77, p = 0.03).

**Table 2:** Sources of Methicillin-Resistant *Staphylococcus aureus* (MRSA) Infection in Year Following Detection of Carriage.

	No. (%) of Infections		
Infection Classification	Total	Pre-Discharge*	Post-Discharge
Total	317 (100%)	132 (100%)	185 (100%)
Pneumonia†	109 (34%)	54 (42%)	55 (29%)
Skin and Soft Tissue†	84 (27%)	25 (19%)	59 (31%)
Primary Bloodstream	56 (18%)	28 (22%)	28 (15%)
Surgical Site	18 (6%)	9 (7%)	9 (5%)
Bone and Joint†	17 (5%)	2 (2%)	15 (8%)
Urinary Tract	10 (3%)	1 (1%)	9 (5%)
Gastrointestinal	7 (2%)	3 (2%)	4 (2%)
Other‡	16 (5%)	10 (8%)	6 (3%)
Associated Bacteremia	82 (26%)	33 (25%)	49 (26%)

* Include infections occurring among outpatients at the time of new detection.

† Differed significantly (p<0.05) between pre- and post-discharge infections using chi-square test.

‡ Includes 6 eye ear, nose, and throat infections, 6 lower respiratory tract infections, 3 cardiovascular system infections, and 1 central nervous system infection.

The risk of subsequent infection during the admission in which MRSA was newly-detected was 14% (82/576). Among those surviving to discharge, the risk of post-discharge MRSA infection was 24% (114/482). These 114 patients had a total of 185 post-discharge infections. The mean time to first post-discharge MRSA infection from the time of new detection was 97 days (median, 72 days). Over half (55%, 104/185) of post-discharge MRSA infections occurred more than 90 days after discharge, and 45 (25%) occurred more than 180 days after discharge. Overall, 21 (11%) post-discharge MRSA infections would have been missed if surveillance was not extended to hospitals other than the one at which MRSA detection was first noted. Furthermore, 99 (54%) were the cause of hospital readmission and an additional 19 (10%) were present upon hospital readmission. Among post-discharge MRSA infections associated with hospital readmission, 97 (82%) were to the same institution in which MRSA was newly-detected.

The distribution of pre- and post-discharge infections among patients newly-detected to harbor MRSA is shown in Table 2. The proportions of skin and soft tissue and bone and joint infections were significantly greater among post-discharge infections compared to pre-discharge infections (p<0.05). In contrast, there was no difference in the proportion of MRSA infections involving bacteremia between pre- and post-discharge infections (χ^2^ = 0.01, p = 0.92). There was also no difference in the risk of post-discharge infection according to whether patients were discharged home (25%, 48/195), to a rehabilitation or skilled nursing facility (23%, 63/274), or to another hospital (23%, 3/13) (χ^2^ = 0.17, p = 0.92).

The vast majority of patients (85%) were re-hospitalized in the year following detection with MRSA (Table 3). Among patients surviving to discharge, 421 (87%) had a total of 732 subsequent hospitalizations. Over one-fourth of patients surviving to discharge (27%, 132/482) had a subsequent hospitalization involving an ICU stay. The mean length of stay among the 99 resultant hospitalizations due to MRSA was 13 days (median, 9 days).

**Table 3:** Post-Discharge Healthcare Utilization in Year Following Detection with Methicillin-Resistant *Staphylococcus aureus* (MRSA).

Source of Post-Discharge Healthcare Utilization*	No. (%) of Patients(N = 498)
Any Hospitalization	421 (85%)
Acute Care Hospital	340 (68%)
Intensive Care Unit Stay	132 (27%)
Emergency Department Visit	319 (64%)
Skilled Nursing or Rehabilitation Facility	301 (60%)
Non-Surgical Procedures	285 (57%)
Surgery	243 (49%)
None of the Above	44 (9%)
Outpatient Visit	394 (79%)

*Proportion of inpatients who survived to discharge from admission in which MRSA was newly-detected. All proportions account for attrition of study population due to deaths in year following detection of carriage.

**Figure 1:**
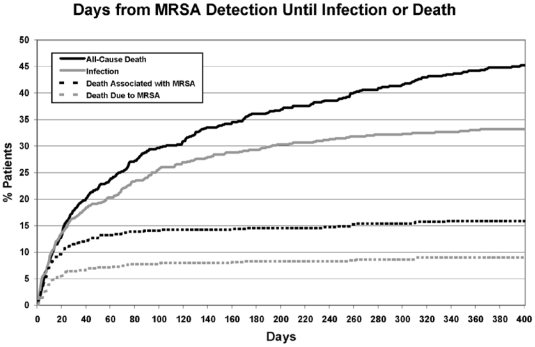
Time from new detection of methicillin-resistant *Staphylococcus aureus* (MRSA) to infection or death.

**Figure 2:**
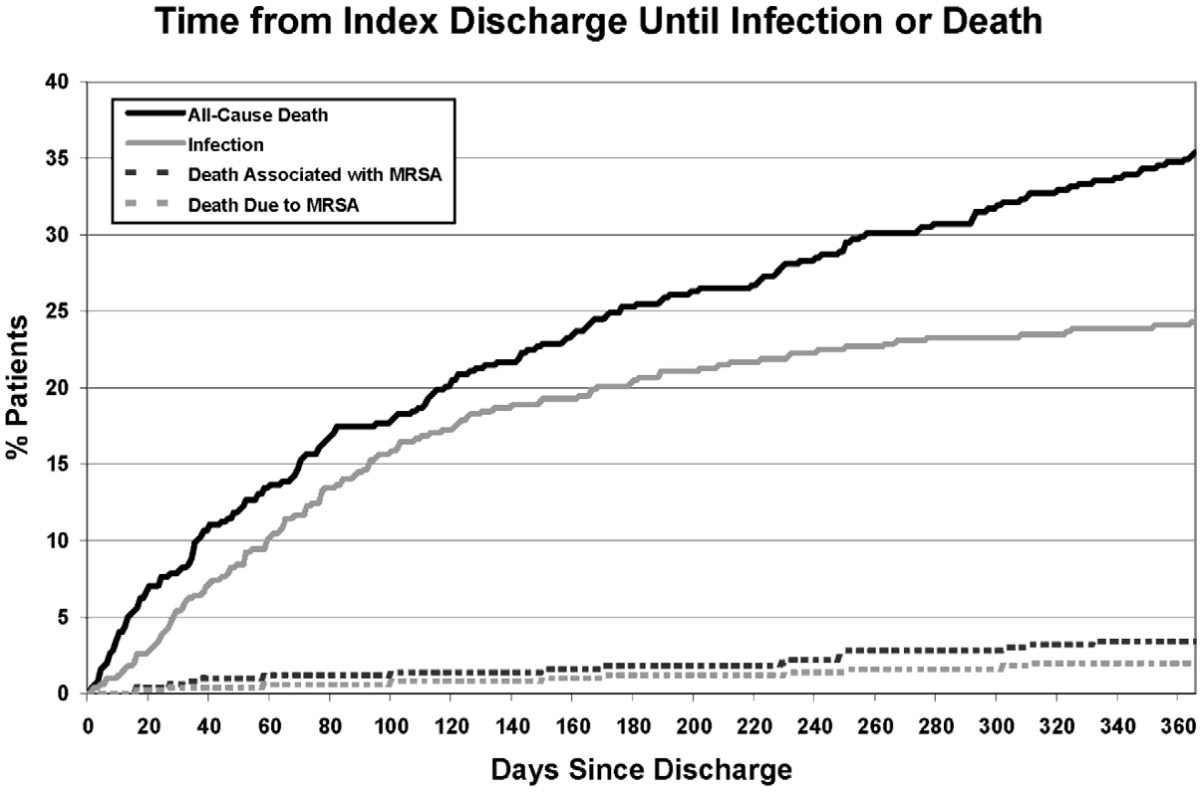
Time to infection or death following discharge from the hospitalization in which methicillin-resistant *Staphylococcus aureus* (MRSA) was newly-detected.

Within one year of MRSA acquisition, death occurred in 46% (269/591) of patients. Over one-third of all deaths (35%, 94/269) were associated with MRSA, and 20% (54/269) were due to MRSA. This translated into a 9.1% risk of death due to MRSA among newly-detected carriers in this cohort. Among those developing MRSA infection, 48% (94/196) died with MRSA infection, and 28% (54/196) died due to MRSA infection. The time to death from the time of detection (Figure 1) and discharge (Figure 2) are shown. There was no difference in the risk of death due to MRSA according to whether patients were discharged home (3%, 5/195), to rehabilitation or skilled nursing facilities (3%, 7/274), or to another hospital (0%, 0/13) (χ^2^ = 0.14, p = 0.93). In comparison, overall annual mortality from Brigham and Women's Hospital excluding obstetric patients ranged from 1.2–2.0% during the study period.

In logistic regression modeling, male gender and age <35 and ≥85 years of age were predictive of subsequent infection with MRSA (Table 4). The only non-surgical procedures significantly associated with subsequent MRSA infection were central venous catheterization and bronchoscopy. The initial site of MRSA detection, initial infection versus colonization, and comorbidities other than diabetes, end-stage renal disease, and end-stage liver disease did not impact the risk of MRSA sequelae.

**Table 4:** Variables associated with Methicillin-Resistant *Staphylococcus aureus* (MRSA) Infection in the Year Following Detection using Multivariate Logistic Regression Testing.

Variable	Odds Ratio (95% CI)	P-value
**Male Gender**	1.3 (1.0, 1.8)	0.05
**Age**		
18–34	2.1 (1.0, 4.4)	0.05
35–44	1.6 (0.8, 3.4)	0.18
45–54	1.0	
55–64	1.3 (0.7, 2.2)	0.37
65–74	1.5 (0.9, 2.5)	0.17
75–84	1.5 (0.9, 2.7)	0.13
≥85	2.5 (1.2, 5.1)	0.02
**Comorbidities**		
Diabetes	1.6 (1.1, 2.2)	0.006
Creatinine ≥2	1.4 (1.0, 1.9)	0.03
Albumin ≤2	1.8 (1.2, 3.0)	0.01
**Non-Surgical Procedures**		
Central Venous Catheter	1.5 (1.1, 2.1)	0.006
Bronchoscopy	1.6 (1.1, 2.5)	0.02

## Discussion

To our knowledge, this work represents the first comprehensive assessment of MRSA infection, hospitalization and death in the year following acquisition in a high-risk tertiary care population. In this cohort, we found that one-third of patients developed subsequent MRSA infection in the year following acquisition. This risk of MRSA infection is substantially higher than those reported in prior studies [1]–[6], including our own work evaluating the risk of subsequent infection based upon medical care delivered solely at the same tertiary care center [13]–[14]. We found that 18% of MRSA infections associated with readmission would have been missed if surveillance was only performed at the same institution as initial detection.

While advances in infection prevention [12] have reduced MRSA healthcare-associated infections, particularly those associated with medical catheters [8]–[9], these results highlight the substantial burden of MRSA that persists after hospital discharge. In this study, nearly 60% of MRSA infections in the year following detection occurred post-discharge. Moreover, the severity of MRSA infections did not abate after hospital discharge with one-quarter of infections involving bacteremia in both pre-discharge and post-discharge periods. These findings provide important opportunities for prevention of readmission and later infection. This is particularly important since hospital length-of-stay continues to decline [7], and increasingly complex medical care is delivered through home health care services, rehabilitation centers, and nursing homes.

Notably, the risk of MRSA infection was not constant over the year following new detection. These data found that infection risk was highest in the 3 months following detection, remained high but to a lesser degree in the next 3 months and then plateaued in the final 6 months of the one-year follow-up. This is consistent with an elevated risk during peri-hospitalization periods, similar to what we previously reported in long-term MRSA carriers [14]. This may be explained by surgeries, procedures, or other health-related events that provide an increased opportunity for invasion, such as through wound-related, device-related or immunologic declines associated with the underlying cause of hospital admission.

We recognize that it is possible, and even likely, that many patients in our cohort acquired MRSA long before detection. Indeed, 40% of patients had an MRSA-positive culture within 2 days of hospital admission, and 81% were hospitalized in the year prior to detection. However, as described above, we believe that MRSA infection risk among carriers may be more strongly linked to hospitalization than time from acquisition. This is supported by the fact that patients do not develop immunity to prior infecting strains of MRSA [17], and that long-term carriers remain at significant risk of MRSA infection [14]. Nevertheless, further research is needed to confirm this belief. Whether or not active efforts to decolonize high-risk patients - either newly detected or persistently colonized with MRSA - are able to prevent later infections is the focus of ongoing clinical trials [18]–[19].

The patients in this study reflect the case mix of a tertiary care academic medical center. It further reflects the case mix of patients who are at risk of MRSA acquisition. The combination of these two is reflected by the very high all-cause mortality one year after MRSA detection. While the risks of subsequent MRSA infection are unlikely to reflect the experience of patients who harbor MRSA in outpatient settings or community hospitals, it highlights the remarkably elevated risk in those who are critically and chronically ill. We postulate that this may reflect the risk of MRSA sequelae not only among tertiary care patient populations, but also among MRSA-positive inpatients with the identified risk factors of advanced age, diabetes, renal insufficiency, or decreased albumin, regardless of hospital type or location. The 33% one-year risk of MRSA infection and the 9% attributable risk of death due to MRSA is concerning in an aging population with rising comorbidities. It is also consistent with the invasiveness of this pathogen. This is not dissimilar to other healthcare-associated pathogens [20], [21] which target high risk groups. One multi-center study of *Clostridium difficile* in predominantly tertiary care hospitals demonstrated a one-month all-cause mortality of 25% and attributable mortality of 7% [20]. Collectively, our work points to the need for interventional studies to reduce the large volume of infections that occur across the continuum of pre-discharge and post-discharge medical care.

This study has several limitations. As mentioned above, it is generalizable only to high-risk MRSA carriers or institutions that care for high-risk patients. In that context, these data may still underestimate infection risk since outpatient MRSA infections were not assessed. In addition, this study describes the risk of subsequent MRSA infections among hospitalized carriers of healthcare-associated strains (known by representative sampling of hospital MRSA strains during this study period). Results may not apply to U.S. hospitals reporting an increasing prevalence of community-associated MRSA strains during this period [22]–[24]. As community-associated strains of MRSA become endemic in healthcare, it remains to be seen whether the spectrum of nosocomial disease and the risk of subsequent infection differs by the origin of MRSA clones. Some evidence has already emerged suggesting that the pathogenesis of community-acquired MRSA differs from healthcare-associated MRSA [25]. Finally, this 13-year cohort may be affected by improvements in medical care and reductions in mortality over the study period. Nevertheless, we note that the risk of MRSA infection among carriers remained stable regardless of the year of cohort entry.

In summary, we show that commercially-insured patients from a tertiary care medical center incur substantial risks of MRSA morbidity and mortality in the year following detection of carriage. One in three MRSA carriers developed MRSA infection, and one in eleven died because of MRSA infection. Nearly 60% of MRSA sequelae occurred after discharge from the hospitalization in which MRSA was newly-detected, and readmission due to MRSA infection was common. These results suggest that preventative interventions seeking to reduce MRSA morbidity and mortality should target patients newly-detected with MRSA and perform post-discharge follow-up across healthcare facilities to account for the substantial risks observed in the period after hospital discharge.
